# Recommendations to the government following the declaration of COVID-19 pandemic

**DOI:** 10.4178/epih.e2020022

**Published:** 2020-04-29

On March 11, 2020, the World Health Organization declared a COVID-19 pandemic. Now, the COVID-19 pandemic must be viewed from a global perspective. Thanks to the efforts of the quarantine authorities, health care workers and the public, the disease is fortunately being controlled in Daegu and Gyeongbuk. However, as sporadic outbreaks in other regions continue to occur, and the risks of epidemics in many countries around the world are increasing, we need to strengthen our boundaries to prevent influx and spread. The goal of quarantine is to slow the spread of disease to a level that our society and the medical system can handle, and to improve the care of critically ill patients to minimize the public’s health damage, including fatality.

In preparation for the prolongation of the COVID-19 pandemic, we recommend that the government strengthen the following:

First, we recommend that the government’s support system to be firmly established around the Prime Minister’s Office so that the Center for Disease Control and Prevention can become the actual top-level department in response to quarantine. In the pandemic phase, the government should focus on policies to minimize the harm of our people. Please mobilize all government departments, local government health centers, public medical centers and administrative competences to ensure that the quarantine policy of the Ministry of Diseases and Disease Control is implemented in a timely manner.

Second, instead of lowering the level of vigilance, the central government should strongly promote ‘social distancing’. In order to minimize transmission in the absence of vaccines and treatments, it is most important that all citizens practice a wide range of ‘social distancing’ simultaneously. Please promote a strong policy that allows the participation of local governments and a wide range of civil society and religious groups at the same time. The government should immediately provide all administrative support and convenience, including economic support, so that our society can withstand such changes without shock. It is advisable to extend school closures to protect students who may become epicenters of community transmission, even if the risk of severe infections is low. Before schools can start again, a response manual for cases of infection in schools, as well as private education facilities such as after school classes, and PC rooms, must be prepared along with measures to keep social distance regarding education and leisure activities. For the safety of collective living facilities such as nursing homes, which are vulnerable to infectious diseases, please prepare proactively.

Third, in order to prepare for large-number of patients and critically ill patients, we recommend that the medical system and the medical delivery system be reorganized. It is possible that there will be multiple cases occurring at the same time anywhere in the country and progressing severely. There is still an urgent need to secure additional hospital beds and isolation facilities for treatment. In order to reduce the fatality rate, please secure medical staffs capable of treating seriously ill patients, expand hospital beds, and establish a power supply system. Convert more public medical institutions into dedicated hospitals and give more support to volunteering private hospitals. As many patients are admitted to the community treatment centers established urgently, number of problems regarding health and safety of patients and workers are arising. Please manage all confirmed problems and establish a manager in charge of community treatment centers, a standard operating procedures, operation manuals, and checklists to ensure safe treatment. It is efficient to categorize the services of public health centers and medical institutions into screening, fever-respiratory care, mild patient care, intensive care, and non COVID-19 patient care to use medical resources in the long-term. Institutional support is needed for a system in which patient transportation to community treatment centers, mild-patient treatment centers, and intensive care centers is harmonious in both directions.

Fourth, active and prompt support for medical institutions is necessary. For the health and safety of the Korean people, policies to prevent the loss of function of medical staffs and medical institutions, who are at the front lines, are necessary. To secure the safety of non-COVID19 patients and staffs, medical institutions require a lot of personal protective equipment, including facial masks. Please provide support promptly so that there is no practical difficulty in securing personal protective equipment. In addition to masks, there is also a need for inspection of medical supplies that require management at a national level. We also recommend initiating a policy for supporting the localization of personal protective equipment.

Fifth, prepare for rational and sustainable anti-disaster measures through interim inspection on the anti-disaster measures so far. In the absence of experience and knowledge of COVID-19, it is time to evaluate and analyze the effectiveness of the quarantine measures implemented. For the implementation of evidence-based preventive measures, an integrated epidemiological information system and a patient information collection system must be promptly established. Effective and appropriate measures must be prepared by inspecting current measures that need to be stopped or modified.

Successful quarantine is minimizing the health damage to our society, even if the overall epidemic period becomes longer. We ask the government to look at this epidemic with long breathing, not to lower the level of vigilance, and to build an effective system to deal with it.

Recommendations to the Public Following the Declaration Of COVID-19 Pandemic

On March 11, 2020, the World Health Organization declared a COVID-19 pandemic. Now, the COVID19 pandemic must be viewed from a global perspective. Thanks to the efforts of the health care workers of the quarantine authorities and the people the disease is fortunately being controlled in Daegu and Gyeongbuk. However, as sporadic outbreaks in other regions continue to rise, and the risks of epidemics in many countries around the world are increasing, we need to strengthen our boundaries to prevent influx and spread. The goal of quarantine is to slow down the disease transmission and to minimize the public’s health damage, including fatality, to a level that our society and the medical system can handle.

Because of the epidemic that has lasted well over a month, many of you will be tired and struggling. However, COVID-19 cannot be controlled by government defense alone.

In preparation for the prolongation of the COVID 19 pandemic, we encourage the public for the following:

First, keep putting your energy into personal hygiene and social distancing. Please refrain from going out and meetings whenever possible. If you need to go to work because it is difficult to work at home or work in rotations, please keep a distance of 2m in work and society. For items shared by several people, wipe the parts you touch with disinfectants. The active participation and efforts of the public will lower the risk of COVID19 transmission and change the epidemic of the disease.

Second, if you have respiratory symptoms such as coughing, refrain from going out and visiting facilities used by many people. The most important way to protect a multi-use facility is for the patient to not to use it. In addition, if you are a person with an old age or have underlying diseases, it is safe to get medical care promptly before progressing to severe illness.

Third, when using a medical institution, be sure to accurately inform your medical staff of your information. If COVID19 spreads in medical institutions, it will interfere with the treatment of the people. To prevent this, medical staffs must accurately know about the information of a patient. This is also necessary for you to be safely and reliably diagnosed and treated.

Fourth, the fact that you have COVID 19 should not be a stigma. Anyone can get COVID 19. When condemned for the fact that a patient has COVID19, patients still suffer from severe psychological sequelae even after overcoming the disease. If patients who need to be diagnosed refuse to be tested because they are afraid of social accusations, it can lead to a greater spread, and we can all suffer.

The end of this pandemic is not likely to come soon. However, if we all respond calmly with one mine, we can minimize the damage to our society. Please help to overcome this crisis wisely. Your efforts can change the epidemic of this disease!

March, 15, 2020

The Korean Society of Infectious Diseases, Korean Association of Infection Control Nurses, The Korean Academy of Tuberculosis and Respiratory Diseases, The Korean Society of Pediatric Infectious Diseases, The Korean Society for Preventive Medicine, The Korean Society of Emergency Medicine, Korean Society for Healthcare-associated Infection Control and Prevention, The Korean Society for Clinical Microbiology, The Korean Society of Critical Care Medicine, Korean Society for Antimicrobial Therapy, Korean Society of Epidemiology

