# Supplementary material for: Recommendations to the government following the declaration of COVID-19 pandemic
Source: Epidemiol Health. 2020 Apr 29;42:e2020022. doi: 10.4178/epih.e2020022 (PMC7285422; doi:10.4178/epih.e2020022)
Supplement: Supplementary file 1 [file epih-42-e2020022-suppl.pdf]

Volume: 42, Article ID: e2020022

https://doi.org/10.4178/epih.e2020022

코로나19 대유행 선언에 따른 대정부 권고안

2020년 3월 11일 세계보건기구는 코로나19(COVID-19) 대유행(pandemic)을 선언했습니다. 이제 코로나19 유행은 전 세계적인 관점에서 보아야 합니다. 방역당국, 보건의료인, 그리고 국민의 노력으로 대구·경북 지역에서는 다행히 조절되어가고 있습니다. 그러나 다른 지역의 산발적인 집단 발생이 지속되고 있을 뿐 아니라 세계 여러 국가의 유행으로 인한 위험이 증가하고 있으므로 유입과 전파를 차단하기 위해 경계를 더 강화해야 합니다. 방역의 목표는 우리 사회와 의료시스템이 감당할 수 있는 수준으로 질환의 전파 속도를 늦추고 중환자 치료를 개선하여 치명률을 비롯한 국민의 건강피해를 최소화하는 것입니다.

코로나19 대유행의 장기화에 대비하여 정부에게 다음을 강화해주기를 권고합니다.

첫째, 질병관리본부가 방역대응의 실질적인 최상위 부서가 될 수 있도록 총리실을 중심으로 정부의 지원체계를 확고하게 구축하기 바랍니다. 대유행 단계에는 정부는 우리 국민의 피해를 최소화하기 위한 정책에 집중해야 합니다. 정부 부처, 지자체의 보건소, 공공의료원과 행정적인 역량을 총동원하여 주무부처인 질병관리본부의 방역 정책이 적시 적소에서 이루어지도록 확실하게 조율해주기 바랍니다.

둘째, 경계의 수준을 낮추지 말고 '사회적 거리두기'를 중앙정부차원에서 강력 추진하기 바랍니다. 백신과 치료제가 없는 상황에서 전파를 최소화하기 위해서는 전 국민이 모두 같은 기간 동안 폭넓은 '사회적 거리두기'를 실천하는 것이 가장 중요합니다. 각 지자체와 광범위한 시민사회, 종교단체의 참여가 동시에 이루어질 수 있는 강력한 정책을 추진해주기 바랍니다. 정부는 우리 사회가 그러한 변화를 충격 없이 잘 버텨낼 수 있도록 경제적 지원을 포함하여 모든 행정적인 지원과 편리를 즉각 제공해야 합니다. 중증 감염의 위험은 낮더라도 지역사회 전파의 역학적 중심(epicenter)이 될 수 있는 학생들을 보호하기 위해서 휴교를 연장하는 것을 권고합니다. 학교뿐 아니라 방과 후 학원, PC방 등 사교육이나 청소년 여가 활동에서도 사회적 거리두기에 대한 방안과 감염자가 발생했을 경우에 대한 대응 매뉴얼을 준비한 후에 개학을 준비하기 바랍니다. 감염병 취약시설인 요양병원, 요양원 등 집단 거주시설의 안전을 위해서 선제적으로 대비하기 바랍니다.

셋째, 대규모 환자와 중환자 발생 가능성에 대비하여 의료시스템과 의료전달체계를 정비하기 바랍니다. 전국 어디에서든 동시에 다수의 환자가 발생하고 중증으로 진행할 가능성이 있습니다. 치료를 위한 병상과 격리시설을 추가 확보하는 것이 여전히 시급하게 필요합니다. 치명률 감소를 위해 중증환자를 진료할 수 있는 의료진을 확보하고 병상 확대와 전원시스템을 확립해 주기 바랍니다. 더 많은 공공의료기관을

Volume: 42, Article ID: e2020022

https://doi.org/10.4178/epih.e2020022

전담병원으로 전환하고 자원하는 민간병원에는 충분한 지원을 해주십시오. 급히 조성된 생활치료센터에 많은 환자가 동시 입소하여 환자와 근무자의 건강과 안전의 문제가 확인되고 있습니다. 제반 문제점을 개선하고 생활치료센터의 운영책임자, 운영표준모델, 운영매뉴얼, 체크리스트 등을 확립하여 안전한 치료를 보장해주십시오. 보건소나 의료기관의 진료업무는 선별진료/발열호흡기진료/경증환자진료/중환자진료/비코로나19환자진료 등으로 구분하는 것이 장기적으로 의료자원을 효율적으로 사용하는 방법입니다. 생활치료센터, 경증치료기관, 중환자치료기관으로의 환자 이송이 양방향으로 원활하게 이루어지는 시스템을 위한 제도적 보완이 필요합니다.

넷째, 의료기관에 대한 적극적이고 신속한 지원이 필요합니다. 국민의 건강과 안전을 위해서 최전선에 있는 의료진과 의료기관의 기능 손실이 발생하지 않도록 보존하는 정책이 필요합니다. 의료기관에서는 코로나19 이외 환자와 의료진의 안전을 위해서 마스크를 포함한 개인보호구가 많이 필요합니다. 개인보호구의 확보에 실질적인 어려움이 없도록 신속히 지원해주기 바랍니다. 마스크 외에 추가적으로 국가적 관리가 필요한 의료물품에 대한 점검도 필요합니다. 개인보호구의 국산화를 위한 지원 정책도 시작하기 바랍니다.

다섯째, 지금까지의 방역대책에 대한 중간 점검을 통해 합리적이고 지속 가능한 방역 대책을 준비하기 바랍니다. 코로나19에 대한 경험과 지식이 없는 상황에서 시행했던 방역대책의 효과를 평가 분석할 시기가 되었습니다. 이를 기반으로 근거기반 예방적 방역조치의 시행을 위해 통합적 역학정보체계 및 환자정보수집체계가 신속히 구축되어야 합니다, 기존의 방역조치에서 중단이나 수정이 필요한 사항을 점검하여 효율적이고 적절한 방역대책을 준비하기 바랍니다.

성공적인 방역은 전체 유행기간이 더 길어지더라도 우리 사회가 입는 건강 피해를 최소화하는 것입니다. **긴 호흡으로 이 유행 상황을 보고 경계의 수준을 낮추지 말고 이 상황에 대처하기 위한 효과적인 시스템을 구축할 것을 정부에게 요청합니다.**

코로나19 대유행 선언에 따른 대국민 권고안

2020년 3월 11일 세계보건기구는 코로나19(COVID-19) 대유행(pandemic)을 선언했습니다. 이제 코로나19 유행은 전 세계적인 관점에서 보아야 합니다. 방역당국 보건의료인, 그리고 국민의 노력으로 대구 경북 지역에서는 다행히 컨트롤되어가고 있습니다. 그러나 다른 지역의 산발적인 집단 발생이 지속되고 있을 뿐 아니라 세계 여러 국가의 유행으로 인한 위험이 증가하고 있어서 유입과 전파를 차단하기 위해 경계를 더 강화해야 합니다. 방역의 목표는 우리 사회와

Volume: 42, Article ID: e2020022

https://doi.org/10.4178/epih.e2020022

의료시스템이 감당할 수 있는 수준으로 질환의 전파 속도를 늦추고 치명률을 비롯한 국민의 건강피해를 최소화하는 것입니다.

이미 한 달을 훌쩍 넘어서 지속되는 유행 상황으로 인해 국민 여러분께서도 많이 지치고 힘드실 것입니다. 그러나 코로나19는 정부의 방역만으로 통제될 수 없습니다.

코로나19 대유행의 장기화에 대비하여 국민들에게 다음에 더 힘써 주시기를 권고합니다.

첫째, 개인위생, 사회적 거리두기에 계속 힘을 쏟아 주십시오. 가능한 외출과 모임을 자제해주시기 바랍니다. 재택근무, 순환 근무 등이 어려워 출근해야 하는 경우 직장과 사회에서 2m 거리 두기에 참여해주십시오. 여럿이 공동으로 사용하는 물품은 손으로 만지는 부분을 소독제로 닦아 주십시오. 국민 여러분의 적극적인 참여와 노력이 코로나19의 전파 위험을 낮추고 이 질환의 유행 양상을 변화시킬 것입니다.

둘째, 기침과 같은 호흡기 증상이 발생한 분들은 외출을 삼가고 여러 사람이 이용하는 시설은 방문하지 않도록 해주십시오. 다중이용시설을 보호하는 가장 중요한 방법은 환자가 그 시설을 이용하지 않는 것입니다. 더불어 연세가 많거나 기저질환이 있는 분이라면 중증으로 진행하기 전에 신속하게 진료를 받는 것이 안전합니다.

셋째, 의료기관을 이용할 때 의료진에게 본인의 정보를 정확하게 알려주십시오. 의료기관 내에서 코로나19가 확산되면 국민 여러분의 진료에 지장이 있게 됩니다. 이를 막으려면 의료진이 각 환자분의 상황을 정확하게 알고 있어야 합니다. 본인이 안전하고 확실하게 진단받고 진료받기 위해서도 필요합니다.

넷째, 코로나19에 걸렸다는 사실이 낙인이 되어서는 안 됩니다. 코로나19는 누구나 걸릴 수 있습니다. 코로나19에 걸린 사실로 인해 비난을 받게 되면 환자들은 이 질환을 극복한 후에도 심각한 정신적 후유증에 시달리게 됩니다. 사회적 비난을 두려워하여 진단을 받아야 할 환자들이 검사를 거부하면 더 큰 확산으로 이어져서 우리 모두가 피해를 볼 수도 있습니다.

이 유행의 끝은 조기에 오지 않을 가능성이 높습니다. 하지만 우리 모두가 한 마음으로 차분하게 대응하면 우리 사회가 입게 될 피해를 최소화시킬 수 있습니다. 이 위기를 슬기롭게 극복해 나갈 수 있도록 국민 여러분께서 힘을 모아 주십시오. **국민 여러분의 노력이 이 질환 의 유행 양상을 변화시킬 수 있습니다!**

2020년 3월 15일

Volume: 42, Article ID: e2020022

https://doi.org/10.4178/epih.e2020022

대한감염학회 대한감염관리간호사회 대한결핵및호흡기학회 대한소아감염학회
대한예방의학회 대한응급의학회 대한의료관련감염관리학회
대한임상미생물학회 대한중환자의학회 대한항균요법학회 한국역학회
